# Parental Perspectives on Paediatric Dental Treatment under General Anaesthesia

**DOI:** 10.3290/j.ohpd.b5569239

**Published:** 2024-07-11

**Authors:** Amal M. Albalooshy

**Affiliations:** Assistant Professor in Paediatric Dentistry, Department of Preventive Dentistry, Faculty of Dentistry, Taif University, Taif, Saudi Arabia.

**Keywords:** dental treatment, general anaesthesia, paediatric, parental

## Abstract

**Purpose:**

To investigate parental perceptions of comprehensive dental care under general anesthesia for their children.

**Materials and Methods:**

The study included parents of children who underwent comprehensive dental care under general anesthesia. Only parents who could communicate in English were included. They were invited to participate in a telephone interview within four weeks of their children’s dental treatment under general anesthesia. The interviews were designed to gather information on three main domains: problems experienced before the operation, children’s well-being after the operation, and satisfaction.

**Results:**

A total of 45 parents participated in the study; 91.1% identified as women and 8.8% as men. Most parents resided in areas categorised as either more deprived (51%) or most deprived (24.4%), based on deprivation indices. Prior to surgery, 66.7% of children suffered from dental pain, 44.4% were affected by dental abscesses or facial swelling, 42.2% experienced difficulties with eating and drinking, while 37.8% experienced sleeping difficulties. Painkillers were used for a short duration to manage post-operative pain (48.9%). Four weeks after the operation, many parents reported improvements in their children’s mouth comfort. They observed positive changes in their children’s ability to eat (40%), sleep habits (33.3%), and overall health and well-being (82.2%). Overall, most parents expressed high levels of satisfaction with the care their children received (95.5%).

**Conclusion:**

Parents observed improvements in their children’s oral health and reported high level of satisfaction with the procedures.

Comprehensive dental care under general anaesthesia (GA) is an important treatment option for children who are unable to cooperate with conventional dental treatments. This may be due to a variety of factors, such as young age, limited cognitive ability, severe anxiety or fear, complexity of dental treatment, and medical status, including special needs in children with complicated medical or developmental conditions.^17^

The most common reasons for dental treatment under GA in children are multiple caries, severe pulpitis necessitating immediate treatment, acute soft-tissue swelling requiring surgical drainage, presence of symptomatic teeth in more than one quadrant, single or multiple dental extractions, traumatic extraction such as ankylosis or fractured tooth, surgical removal or exposure of unerupted tooth, soft or hard tissue biopsy, wound debridement, post-operative hemorrhage necessitating packing and suturing, and intraoral examination with radiographs under GA for children with special needs who have dental problems that require treatment under GA.^8^

Some previous studies have explored parental perceptions of children’s comprehensive treatment under general anaesthesia. These studies provide valuable insights into overall health, oral health-related quality of life, satisfaction, concerns and complications.^2,3,22^ Common post-operative complications include pain, bleeding, sore throat, fever, nausea, vomiting, weakness, dizziness and sleepiness.^3^ In instances in which complications arising from the operation are minimal, parental satisfaction tends to increase.^6^ However, these studies did not capture the nuances of parents’ perceptions because they relied on closed-ended questionnaires instead of interviews.

Understanding parental perceptions of comprehensive dental care under general anaesthesia is essential for developing patient-centered oral healthcare strategies. By exploring parental perspectives, researchers and dental professionals can gain valuable insights into parental satisfaction and potential areas of improvement to enhance the quality of dental care delivery and to better meet the needs and expectations of parents and their children.^23^

Therefore, this study aimed to investigate the perceptions of parents regarding comprehensive dental care under general anaesthesia for their children at the Leeds Dental Institute.

## Materials and Methods

This study was reviewed and approved by the Leeds Teaching Hospitals NHS (LTH/AFS/0149). The participants were identified through the general anaesthetics database of children scheduled for comprehensive dental treatment at the Paediatric Dentistry Department of the Leeds Dental Institute, Leeds, UK. Written informed consent was obtained from parents who agreed to take part in the study..

A purposive sampling technique was employed in this study. Participants were chosen based on specific characteristics relevant to the research topic.^7^ It was used to enable a comprehensive understanding of the research question by the generated themes. Recruitment continued until data saturation was reached.^24^ In order to promote sample diversity, participants were intentionally selected from various sociodemographic backgrounds to ensure representation. Furthermore, their children presented with distinct issues prior to the procedure, ensuring a broad range of challenges was represented.

The study included parents of children who had undergone comprehensive dental care under general anaesthesia between February 2019 and May 2019. This dental care was provided by National Health Service (NHS) in the UK free of charge for children under 16 years of age. The children were referred from general dentists or paediatric dentistry specialists who did not have general anaesthesia facilities at their practice.

The inclusion criteria required the participants to communicate in English. To ensure informed consent, the parents were provided with two information sheets. The first information sheet was sent to the patients’ home address prior to dental treatment, along with a consent form. The second information sheet was administered on the day of dental treatment, either after the initial assessment or after providing post-operative instructions, and written consent was obtained.

Within four weeks of dental treatment under general anaesthesia, the author approached the parents and invited them to participate in a semi-structured interview conducted over the telephone. This interview was designed to gather information and insights into the experiences and perspectives of parents regarding the dental treatment that their child had received.

During the telephone interviews, the questions focused on three primary domains: problems experienced before the operation, the child’s postoperative well-being, satisfaction levels, and meeting expectations.

Before conducting the actual interviews with participants, a pilot study including 10 parents was conducted to test and refine the interview protocol. The interviewer followed the semi-structured interview protocol consisting of a set of predetermined questions. The participants were encouraged to provide feedback on the clarity and relevance of the questions, as well as any difficulties they encountered in understanding or responding to them.

The pilot study revealed some areas where improvements could be made to enhance the interview protocol. Participants expressed some confusion regarding the wording of certain questions, which were subsequently modified to improve clarity. Additionally, the flow of the interview was adjusted to allow for more flexibility in exploring participants’ responses.

The data collected from the interviews were entered into SPSS version 27 (SPSS; Chicago, IL, USA) using a unique identification number assigned to each participant. To ensure data security, the information was saved on a password-protected university computer, which provided a secure environment for data analysis.

## Results

Of the 60 parents approached for telephone interviews, 45 agreed to participate. Among the participants, 41 (91.1%) were women, while four (8.8%) were men. The majority of parents were between the ages of 36 and 45 years. In terms of ethnicity, the most common group was white, accounting for 24 (53.3%) participants, followed by Asian/British with 9 (20%) participants. When considering deprivation levels, most parents were classified as either more deprived (n = 23, 51%) or most deprived (n = 11, 24.4%) (Table 1).

**Table 1:** Parenteral sociodemographic characteristics (n = 45)

Characteristics	Frequency
**Gender**	
Female	41
Male	4
**Age group (years)**	
25–30	3
31–35	7
36–40	22
41–45	13
**Ethnicity**	
White	24
British	4
Asian	3
Asian/British	9
Black	3
Mixed	2
**Levels of de privation**	
Most deprived	11
More deprived	23
Average	7
Less deprived	3
Least deprived	1

Overall, all the parents expressed that the information they provided was easy to understand. However, a small percentage of parents (n = 3, 6.6%) found the information unclear and difficult to comprehend.

### Theme 1: Problems Experienced before the Operation

Before surgery, a significant number of children experienced various dental issues. Out of the total sample, 30 children (66.7%) reported dental pain, while 20 children (44.4%) had dental abscess or facial swelling. Additionally, 16 children (35.6%) were prescribed antibiotics, 19 (42.2%) faced challenges with eating and drinking, and 17 (37.8%) experienced difficulty sleeping (Table 2).

**Table 2:** Preoperative problems (n = 45)

Problems	Frequency	
	Yes	No
Pain or toothache	30	15
Dental abscess or swelling	20	25
Antibiotics for dental problems	16	29
Difficulty in eating/drinking	19	26
Difficulty in sleeping	15	30

### Theme 2: Patients’ Postoperative Well-being

Almost half of the patients experienced pain immediately after the operation (n = 22; 48.9%). Among the parents of these children, 16 (72.7%) reported that the level of pain was greater than expected, three (13.6%) mentioned that it was less than expected, and three (13.6%) found it to be as expected. The majority of parents (n = 15, 68%) reported that their child experienced pain on the day of the operation, while seven parents (31.8%) reported pain persisting the next day. Pain management primarily involved the use of paracetamol or calpol (n = 13, 59%), with a smaller percentage using ibuprofen or nurofen (n = 2, 9%) or a combination of both (n = 7, 32%). Most patients required painkillers only for a short duration, with 82% (n = 18) taking them for fewer than 3 days. Only four patients (18%) required pain relief for a longer period, specifically 3-7 days.

Furthermore, 11 (24.4%) children experienced post-operative nausea. Only three of these children vomited. Most parents (n = 8, 73%) reported that their children experienced more episodes of illness than anticipated. Two parents (18%) reported the level of illness to be less than expected, whereas one parent (9%) reported it to be as expected.

Only two patients experienced bleeding after the operation and were required to return to the hospital on the same day. However, both patients received appropriate medical attention and remained in the hospital for a period 4-8 h. Following thorough evaluation and management, no further complications occurred, and both patients were discharged to their homes without any additional issues.

Four weeks after surgery, most parents (n = 39, 87%) reported that their children’s mouths felt more comfortable. Only a small percentage (n = 6, 13%) of patients showed no noticeable differences. Approximately half of the parents (n = 25, 55.5%) did not observe any changes in their children’s eating patterns. However, 18 parents (40%) reported that their children’s eating habits improved or became easier after the surgery. Only two parents (4.4%) reported difficulties in eating, possibly because their children had undergone approximately 10 extractions (Table 3).

**Table 3:** Postoperative changes (n = 45)

Question	Response		
Is your child’s mouth more comfortable now?	More comfortable 39	Less comfortable 0	No difference 6
Has your child’s ability to eat/drink changed?	Better/easier 18	Worse/harder 2	No difference 25
Has your child’s sleep changed?	Better/easier 15	Worse 0	No difference 30
Do you think the dental treatment has changed your child’s overall health?	Improved 37	Worse 0	No difference 8
Do you think your child’s overall well-being or general happiness/behaviour has changed?	Improved 37	Worse 0	No difference 8

In terms of changes in children’s sleeping patterns after the operation, most parents (n = 30, 66.7%) reported no statistically significant differences. However, one-third of the parents (n = 15, 33.3%) noted that their children experienced better or easier sleep following dental treatment. Furthermore, a significant majority of the parents (n = 37, 82.2%) believed that dental treatment positively affected their children’s overall health. They observed improvements in their children’s overall well-being, general happiness, and behaviour. Conversely, a small proportion of parents (n = 8, 17.8%) reported no noticeable differences in these aspects (Table 3).

### Theme 3: Satisfaction and Meeting Expectations

When asked about their parents’ satisfaction with the care provided, most reported having a positive experience. Specifically, 38 parents (84.5%) expressed that they were very satisfied, while 5 parents (11.1%) reported being satisfied. These parents indicated that the surgery met their expectations. However, two parents (4.4%) expressed dissatisfaction, primarily because the number of teeth extracted during the procedure exceeded their initial expectations.

Furthermore, when parents were asked about the likelihood of recommending a service to friends and family, their responses varied. Most parents, 31 (68.9%) indicated that they were extremely likely to recommend the service. Additionally, 12 parents (26.7%) stated that they were likely to recommend it, while two parents (4.4%) expressed that they were unlikely to do so. Table 4 provides an overview of parents’ satisfaction levels along with their corresponding responses (quotes). Within the main theme of satisfaction, five subthemes emerged: satisfaction with the caregiver, satisfaction with the dental treatment, satisfaction with the treatment outcome, satisfaction with the service, and dissatisfaction. These subthemes captured various aspects of parents’ satisfaction and provided a deeper understanding of their experiences.

**Table 4:** Parenteral satisfaction level

Sub-theme	Parents’ comments
Satisfaction with the caregiver	“Fantastic treatment ... friendly, very nice and experienced staff.” P6
	“Better dental treatment than GDP, more professional and expert.” * P10
	“Really excellent and helpful staff.” P11
	“Consultant X was brilliant and all staff were very helpful.” P12
	“Staff were fantastic and lovely.” P13
	“Very nice people, looked after my child very well and explained everything.” P16
	“Really nice and helpful staff.” P21
	“Staff are helpful, supportive and looked after us very well.” P22
	“Everything was fine, and staff were very helpful.” P25
	“Team sorted everything quickly.” P31
	“Good experience, I am very pleased as doctors gave lots and lots of information about my child’s needs.” P33
	“Staff explained everything very well.” P36
	“My son is a special need child…. they were very great with him.” P37
	“Staff treated my child very well, they were patient with my child, great work and everything was fantastic.” P38
	“Staff looked after my child very well, explained everything well.” P 41
	“Everybody was nice, good explanation for everything, I was scared at the beginning, but the staff reassured me.” P42
	“Fantastic and brilliant team. We did not have any problems.” P43
	“I really appreciate the team efforts …. they did a fantastic job.” P44
Satisfaction with the dental treatment	“The care was excellent.” P23
	“Everything was perfect.” P24
	“Happy with the treatment, everything was fine.” P27
	“We kept up to date, everything well explained and excellent care.” P28
	“Good care.” P29
	“Looked after my child well.” P30
	“Treatment was fine, they treated my child with respect, and I really appreciated it.” P35
	“Everything went perfect. My child did not suffer after GA.” P36
	“My child’s is improved. He is a very happy boy with no pain.” P41
Satisfaction with outcome of the treatment	“Settled mouth and better life.” P1
	“My child treated very well, and his health improved a lot.” P3
	“I am very happy with the results.” P11
	“We achieved everything we want.” P17
	“Got everything sorted.” P21
	“My child’s health is improved.” P24
	“My child is happier and more comfortable.” P44
Satisfaction with service	“Everything was ok, the service was good, and my child is better.” P4
	“Everything was perfect and good care overall.” P7
	“Good service and care, everything was good.” P9
	“Overall excellent service.” P12
	“We did not have any problems with the service.” P14
	“We are happy with the given service.” P17
	“Nothing was wrong, everything was good.” P20
	“Great service, it was lovely, and they did the job quickly.” P39
	“Very good service.” P45
Dissatisfaction	“I am not happy .. they pulled out so many teeth .. more than what I expected.” P15
	“I am still upset with the number of extracted teeth. I cannot recommend to service to friends and families as they might have the same problem.” P34

*GDP: general dental practitioner; P: parent.

## Discussion

This study aimed to investigate parents’ perceptions of comprehensive dental care under general anaesthesia for their children at the Leeds Dental Institute. This study emphasised three primary themes: 1) problems before surgery; 2) the child’s post-operative well-being; and 3) satisfaction and meeting expectations.

The present study found that the primary issues reported by parents regarding their children’s condition before the surgery included dental pain, abscesses, and facial swelling requiring repeated antibiotic treatment. These problems negatively affect children’s daily functional abilities, particularly in relation to eating and sleeping. These findings align with those of previous studies in which both parents and children identified pain as the most common complaint and its direct impact on children’s ability to eat and sleep.^5,12,19-21^

A recent study in Saudi Arabia involving 319 parents revealed that parents were concerned regarding potential complications associated with general anaesthesia (78%), followed by postoperative pain (51%), use of intravenous lines and cannulas (49%), and the possibility of coma or death (46%).^2^ Hence, it is crucial to consider these concerns before administering general anaesthesia.

In the current study, a significant number of parents reported that their children experienced pain primarily on the day of the procedure. However, the pain was effectively managed by the administration of painkillers. Additionally, a small number of children felt sick and only a few experienced vomiting. Parents reported that their level of pain and sickness exceeded their initial expectations. These findings provide valuable insights into the range of issues that children may experience after GA, thereby helping healthcare providers anticipate and address these concerns to provide optimal post-operative care. Understanding specific complaints and their frequency enables healthcare professionals to tailor interventions and support to meet the individual needs of children during recovery.

However, Farsi et al^11^ reported that 99% of children experienced one or more complaints on the first post-operative day, whereas only 33% complained by the third day. In their study, the most prevalent complaints on the first day were difficulty eating (86%), sleepiness (71%), and pain (48%). Other common complaints included bleeding, drowsiness, sore throat, vomiting, psychological changes, fever, coughing, and nausea.

In our study, parents reported a notable improvement in their children’s well-being following surgery. They observed that their children said their mouths felt more comfortable, and that there was an improvement in eating, that is, it was easier for them to eat. In addition, the parents noticed improvements in their children’s sleep patterns. There was a significant enhancement in their children’s overall happiness and behaviour. These findings align with those of several previous studies that have reported an improvement in the overall quality of life of children receiving comprehensive dental treatment under general anaesthesia (GA). Comprehensive dental treatment appears to have a positive impact on the well-being of children, leading to improved outcomes and an enhanced quality of life.^9,10,13,15,22^

The present study also investigated parental satisfaction. The majority of parents expressed satisfaction, particularly in the following areas: satisfaction with the caregiver, satisfaction with the dental treatment received, satisfaction with the treatment outcomes, and satisfaction with the overall service provided. However, only two parents reported dissatisfaction, primarily because of the significant number of teeth extracted, which negatively impacted their children, particularly during eating.

A recent cross-sectional study involving 306 parents revealed a high level of satisfaction with treatment provided under GA. The study found that an overwhelming majority of parents (95.8 %) were satisfied with the treatment that their children received. This high satisfaction rate reflects the positive experiences and outcomes reported by parents in relation to the healthcare services provided.^6^ Other studies have reported similar findings.^1,4,19^

The current study strengthens the understanding that patients residing in deprived areas bear a significant burden of oral diseases. Hence, this study provides further evidence that individuals in socioeconomically disadvantaged situations face greater challenges and heavier disease burdens than their counterparts in more affluent regions. In a Canadian study, it was observed that oral disorders had a minimal impact on the health-related quality of life of children from higher-income households. However, a statistically significant and noticeable effect was observed among children from low-income households.^16^

The findings of the present study may provide valuable guidance to dental practitioners, policymakers, and researchers for optimising the provision of comprehensive dental care under general anaesthesia. From the parental perspective, stakeholders can work collaboratively to address concerns, improve communication strategies, and develop interventions that enhance the well-being and parental satisfaction of young patients undergoing such procedures.

Nevertheless, it is important to note that most previous studies relied on questionnaires to assess parental satisfaction. While these questionnaires provided valuable insights into overall satisfaction levels, they did not delve into the specific reasons for parental satisfaction. In contrast, our study delved deeper into the reasons for parental satisfaction by conducting interviews.

This study has some limitations. The interviews were caried out within four weeks after the procedure, which may introduce recall bias, as participants may have had difficulty accurately remembering past experiences. Additionally, the study was conducted at a specific location in Leeds, UK, which may limit the generalisability of the results to other geographical areas. Furthermore, the use of proxy reporting, which is valuable for young children, may introduce caregiver burden bias and may not fully capture the child’s perspective.^14,18^ Future research should consider employing child-report measures specifically developed and evaluated for use in young children undergoing dental treatment under general anaesthesia (GA). This provided a more comprehensive understanding of their experiences and outcomes.

## Conclusions

Following a complete oral rehabilitation procedure performed under general anaesthesia, there was noteworthy advancement in oral health. Parents acknowledged a substantial enhancement in both the oral and overall health of their children. Moreover, the satisfaction levels among parents were found to be high with the service provided, caregivers, and treatment and its outcomes.
